# The Acceptability of a Psychoeducation and Skill-Based Training for Carers and Teachers to Cope with Risky Behaviours in Adolescence

**DOI:** 10.3390/children11010038

**Published:** 2023-12-28

**Authors:** Ludovica Natali, Valentina Cardi, Marco Lunghi, Rosanna Ferrara, Linda Marconi, Gioia Bottesi

**Affiliations:** 1 Centro di Ateneo Servizi Clinici Universitari Psicologici, University of Padova, 35131 Padova, Italy; ludovica.natali@phd.unipd.it (L.N.); valentina.cardi@unipd.it (V.C.); marco.lunghi@unipd.it (M.L.); rosanna.ferrara@unipd.it (R.F.); linda.marconi@unipd.it (L.M.); 2 Department of General Psychology, University of Padova, 35131 Padova, Italy; 3 Department of Psychological Medicine, Institute of Psychiatry, Psychology and Neuroscience, King’s College London, London SE5 8AF, UK; 4 Department of Developmental Psychology and Socialisation, University of Padova, 35131 Padova, Italy

**Keywords:** psychoeducation, skills-training, adolescence, risky behaviours

## Abstract

Childhood and adolescence psychopathology is associated with an increased risk of psychological difficulties in adulthood. Early interventions for youth should provide carers and teachers with knowledge and skills to respond to adolescents’ risky behaviours. This study evaluated the acceptability and effectiveness of a single 3-h workshop, combining psychoeducation and skills training to promote knowledge about, and confidence to address, adolescents’ risky behaviours in carers and teachers of adolescents aged 10–14. Demographics and perceived self-efficacy in the parental or teaching role were collected at baseline using self-report questionnaires. Motivation and confidence to respond to adolescents’ risky behaviours were measured before and after the workshop using motivational rulers. Participants provided written feedback about their experience about the workshop. Twenty-seven carers and 27 teachers attended the workshops. Teachers reported a significant increase in both importance (*p* = 0.021) and confidence (*p* < 0.001) to respond to risky behaviours following the workshop. This change was associated with baseline self-efficacy levels (importance: *p* = 0.011; confidence: *p* = 0.002). Carers also reported greater confidence to address risky behaviours following the workshop (*p* = 0.002). Participants found the contents and methods of the workshop highly acceptable. Online and multiple-session workshops might increase reach and effectiveness.

## 1. Introduction

Adolescence is a phase characterized by heightened psychological vulnerability [1]. Approximately 8.8% of children and adolescents worldwide have been diagnosed with a mental disorder [2]. The increased vulnerability to mental health problems in adolescence is partly due to the numerous developmental challenges and tasks that young individuals face in this phase (e.g., search of their own identity, achieving autonomy) [3,4,5]. Furthermore, in recent years, worries and uncertainties about major events, such as the COVID-19 pandemic and climate change, have contributed to increased adolescent vulnerability to psychological distress [6,7,8]. Over time, if not addressed effectively, psychological difficulties early in life can lead to maladaptive outcomes in adulthood, including emotional and interpersonal difficulties, and poor mental health [9,10]. This posits the need for prevention programmes and early interventions to address youth psychological difficulties. A recently formulated roadmap to develop effective early interventions highlights the need for greater exchange of knowledge and skills between professionals and carers [11]. This is because significant others can influence the trajectory of mental health difficulties, either reinforcing these difficulties or providing valuable support to tackle them [12,13,14].

In recent years, an increasing number of studies have explored the effectiveness of single-session interventions (SSIs). SSIs can be defined as “specific, structured programs that intentionally involve just one visit or encounter with a clinic, provider, or program” [15]. SSIs include core components of comprehensive, evidence-based interventions and deliver them succinctly to enhance access and completion rates. As a result, appropriately targeted SSIs can offer a cost-effective supplement or alternative to standard care, particularly when considering that longer interventions do not necessarily equal more effective outcomes [16,17]. So far, single-session parent training has yielded positive outcomes, including improvements in parents’ knowledge, well-being, and self-efficacy [18,19,20].

For this purpose, a single-session workshop for carers and teachers of adolescents aged 10–14 was developed, with the goal of providing them with knowledge and skills to identify and address at-risk behaviours. In particular, the workshop addressed topics such as developmental changes in adolescence and factors which might provide mental health risk or resilience. This study reports on carers’ and teachers’ outcomes of the workshops. The primary aim of the study was to assess the acceptability of the workshops (e.g., the degree of perceived involvement and satisfaction; strengths and weaknesses). Secondary aims were: (1) assessing the short-term impact of the workshops on participants’ importance and confidence to identify and respond to adolescents’ risky behaviours; (2) examining whether this change correlated with participants’ self-efficacy at baseline. Studies on health behaviour indicate that motivation, as well as self-efficacy, mediate the impact of learning on behaviour change [21,22]. Accordingly, changes in motivation were considered as proximal indicators of behaviour change. The hypotheses were that the workshops would have led to greater importance and confidence to identify and respond to risky behaviours in adolescence compared to baseline. An additional goal was to examine the relationship between baseline levels of self-efficacy and changes in motivation.

## 2. Materials and Methods

### 2.1. Participants

The study targeted carers and teachers of adolescents aged 10–14 years. There were no exclusion criteria. Participants were recruited from estate (public) secondary schools in the northeast of Italy. All participants were initially informed about the workshop through informative posters and flyers made available in schools. Particular attention was given to the recruitment of families belonging to ethnic minorities. All carers who participated in the workshop were parents.

### 2.2. Intervention

The intervention consisted of a single 3-h workshop specifically designed for either teachers or carers (i.e., no joint teacher–parent workshops were organised). In total, four workshops were conducted (two for carers, two for teachers) from March 2023 until May 2023 (the project’s duration was 17 months, starting on June 2022). Three expert psychologists (ML, LM, RF) specialized in clinical psychology, developmental psychology, and school psychology facilitated the workshops. All workshops were divided into two parts.

In the first part, participants were provided with psychoeducation about the changes and processes characterizing typical development during adolescence, with particular reference to neurobiological maturation, the development of cognitive and metacognitive abilities, the formation of one’s identity, the salience of peers, and increased academic demands and self-regulation (e.g., [3,4,5,23]). Particular attention was given to how different factors, such as temperament, beliefs, parenting style, scholastic experiences, motivation, intelligence, and locus of control could shape the developmental trajectory (e.g., [24,25,26]).

In the second part of the workshop, participants were encouraged to learn by experience. They were asked to identify and respond to difficult at-risk behaviours by reading and answering questions about a series of illustrative vignettes.

### 2.3. Vignettes

Six illustrative vignettes were developed for the skills-training component of the workshop (three for carers and three for teachers). Each vignette described a scenario aimed at training participants in identifying and responding to risky behaviours in adolescents. For teachers, the vignettes covered the following themes: (1) learning difficulties; (2) ethnic minorities; (3) scholastic overachievement. For carers, the topics included: (1) autonomy and peer influence; (2) scholastic overachievement and risk of isolation; (3) scholastic demands. Examples of vignettes are shown in Table 1. After reading each vignette, participants were divided into groups and asked to answer the questions.

### 2.4. Measures

Before the workshop, participants completed a demographic questionnaire including questions on age, gender, nationality, first language, level of education, marital status, number of children, financial income, psychological difficulties, and medical illnesses. All variables are displayed in Table 2. Participants also completed the following measures:

The Depression, Anxiety, Stress Scale-21 (DASS-21) [27,28] to measure carers’ and teachers’ psychological distress. Items are scored on a 4-point Likert scale, where higher scores indicate greater severity of the symptoms in the last week. The total score demonstrated excellent internal consistency in this study (Cronbach’s Alpha for teachers and carers = 0.9).

The Short Form of the Teachers’ Sense of Efficacy Scale (TSES-SF) [29,30] to assess teachers’ self-efficacy. The scale comprises three subscales (i.e., teacher self-efficacy in student engagement, teacher self-efficacy in instructional strategies, and teacher self-efficacy in classroom management) and a total score. Items are scored on a 9-point Likert scale, where higher scores indicate greater self-efficacy. The total score demonstrated excellent internal consistency in this study (Cronbach’s Alpha = 0.9).

The Parenting Sense of Competence (PSOC) [31] to assess carers’ efficacy in the parenting role. The questionnaire comprises two subscales (i.e., satisfaction and efficiency) and a total score. Items are scored on a 6-point Likert scale, and higher score indicate greater parenting sense of efficacy. There is an Italian version of the PSOC [32] and it has been used in other studies [33,34]. The total score of this instrument demonstrated good internal consistency in this study (Cronbach’s Alpha = 0.8).

The motivational ruler to assess participants’ motivation and confidence to respond to risky behaviours in relation to the situation described in the vignette. Two questions (one for motivation and one for confidence) were designed for each vignette and rated on a Visual Analogue Scale (VAS) from 0 (“Not at all”) to 10 (“Extremely”).

Qualitative feedback form. This evaluation form consisted of 7 items designed to collect participants’ feedback on the workshop’s content and delivery method. Four items were scored on a 4-point Likert scale (0 = “Not at all”-3 = “Very much”). The questions evaluated the clarity of the objectives (i.e., “How clear were the workshop objectives?”), the usefulness of the workshop (i.e., “How useful was the workshop to you?”), the adequacy of the delivery methods (i.e., “In your opinion, were the workshop delivery methods adequate?”), and the level of participation/involvement (i.e., “How engaged did you feel?”). Three questions were open-ended and asked information about the strengths (i.e., “In your opinion, what were the strengths of the workshop?”) and weaknesses of the workshop (i.e., “In your opinion, what were the weaknesses of the workshop?”), as well as if there would have been other topics participants would have liked to discuss (i.e., “Were there any topics you would have liked to discuss during the workshop? If so, which ones?”).

### 2.5. Procedure

Schools interested in the workshops were identified through the collaboration with local parents and teachers organizations (i.e., the Veneto Regional Coordination of School Council Presidents) and the municipal administration of Conegliano. Representatives of each school provided the complete list of carers and teachers interested in attending the workshop. The workshops took place in the school premises. One week prior to the workshop, participants completed an informed consent form and the baseline assessment through the online platform Qualtrics. On the day of the workshop, before and after the session, participants were given the vignettes and asked to complete the motivational ruler. At the end of the workshop, participants also completed the qualitative feedback form.

Ethical approval was obtained by the Ethics Committee for the Psychological Research of the University of Padova (reference number: 5195). The study was conducted in accordance with the Declaration of Helsinki. Written informed consent was provided by all participants.

### 2.6. Data Analysis

Descriptive analyses were used to describe the socio-demographic characteristics of the participants and to evaluate the acceptability of the workshops.

Paired-samples Wilcoxon tests were calculated on the motivational rulers to assess the impact of the workshops on motivation and confidence to address risky behaviours. Global scores for confidence and importance were obtained by averaging participants’ answers to the vignettes. Rank biserial correlations were used to estimate effect sizes (ESs). The coefficient was described as tiny (<0.5), very small (≥0.05 and <0.1), small (≥0.1 and <0.2), medium (≥0.2 and <0.3), large (≥0.3 and <0.4), and very large (≥0.4) [35].

Spearman’s correlations were performed to examine whether changes in motivation and confidence to tackle risky behaviours throughout the workshop would be related to teachers’ and carers’ self-efficacy at baseline. To do so, delta scores for motivation and confidence were computed, with positive scores indicating an improvement.

All the above-mentioned analyses were carried out on JASP [36], and statistical significance was set at *p* < 0.05.

## 3. Results

### 3.1. Socio-Demographic Characteristics

**Figure 1:**
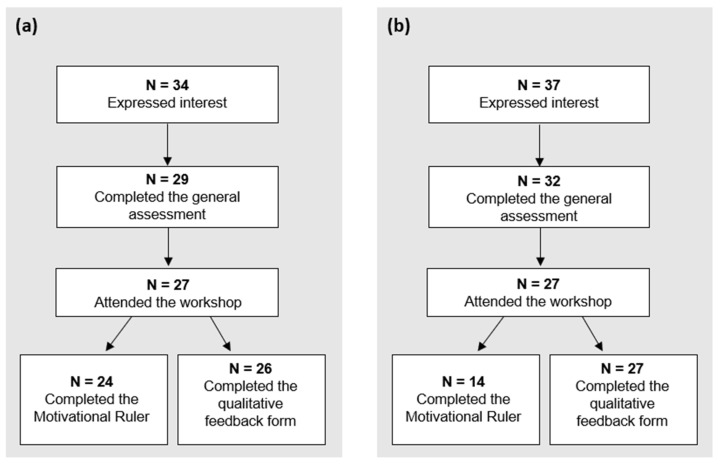
A CONSORT diagram describing the participation to the study of (**a**) teachers and (**b**) carers.

Thirty-four teachers and 37 carers expressed interest in participating the workshop. However, the final sample included 29 teachers and 32 carers, i.e., those who completed at least the baseline questionnaire. The flow of participation in the study is described in Figure 1. Participants’ socio-demographic characteristics are presented in Table 2.

The mean age of teachers was 50.64 (7.35), while the mean age of carers (all parents) was 48.49 (5.54). Most of the sample comprised women (n = 55, 90.16%) and Italian nationals (n = 69, 96.72%). Most participants had a master’s degree (n = 42, 70.00%), were married (n = 37, 62.71%), and were house owners (n = 43, 76.79%). Teachers reported an average number of children of 1.32 (1.07), while carers reported 2.00 (0.62). A slight difference was observed between teachers and carers with regards to income, with teachers reporting overall a smaller income. Only a small percentage of the sample reported experiencing psychological difficulties (n = 8, 13.56%) or medical conditions (n = 7, 11.86%).

### 3.2. Psychological Distress and Self-Efficacy

Most of the sample reported low levels of psychological distress (Teachers: n = 28, 96.55%; Carers: n = 29, 93.55%) with only a small minority reporting high levels (Teachers: n = 1, 3.45%; Carers: n = 2, 6.45%) [27].

Teachers reported lower self-efficacy compared to the normative Italian sample ([29]; *N* = 200; Mean = 7.02; SD = 1.45; t = −2.25, *p* = 0.03). Carers, from an observational perspective (since Italian normative data for comparison were unavailable), reported slightly higher self-efficacy compared to an Italian sample of mothers of children with typical development ([37]: *N* = 240; Mean = 60.47; SD = 10.85).

### 3.3. Workshop Acceptability

Overall, out of the 71 participants who initially expressed their interest in attending the workshop, 54 individuals (76.06%) attended the workshop, with 48 of them (67.61%) completing the motivational ruler and 53 (74.65%) completing the qualitative feedback form. Drop-outs were largely due to organizational difficulties (e.g., delayed start of the workshop, early closure of the school facilities). Descriptives on qualitative feedback are shown in Table 3.

Overall, participants rated the workshop positively: the objectives were clear, the content was perceived as very helpful, they appreciated the delivery methods, and they felt very engaged. Participants felt that working in a group was one of the major strengths. They identified the one-off format of the workshop and the low attendance rate as weaknesses. They also expressed a desire for practical strategies to respond to problematic behaviours. Most teachers reported that they would be interested in learning more about group dynamics within the classroom and strategies for managing them. Carers were mostly interested in exploring the use of digital technologies and social media in adolescence.

### 3.4. Changes in Importance and Confidence to Address Risky Behaviours across the Workshop

Results of the Wilcoxon matched-pairs signed rank tests are reported in Table 4.

A significant increase in teachers’ importance (*p* = 0.021) and confidence (*p* < 0.001) to address risky behaviours was observed, with very large effect sizes. Likewise, carers reported a significant increase in their confidence to tackle risky behaviours after the workshop (*p* = 0.002), with a large effect size. No significant change in importance to respond to risky behaviours was observed in carers (*p* = 0.241).

### 3.5. Relationship between Changes in Motivation and Self-Efficacy

Teachers’ baseline levels of self-efficacy were significantly associated with changes in teachers’ importance (*r*_s_ = −0.51, *p* = 0.011, n = 24) and confidence (*r*_s_ = −0.60, *p* = 0.002, n = 24) to respond to risky behaviours. Specifically, greater improvements in importance and confidence to respond to risky behaviours were associated with lower teaching self-efficacy at baseline.

No significant correlations were found between carers’ self-efficacy and their motivation (*r*_s_ = −0.16, *p* = 0.626, n = 12) and confidence (*r*_s_ = −0.37, *p* = 0.234, n = 12) to address risky behaviours.

## 4. Discussion

This article describes the preliminary findings of a workshop combining psychoeducation and skills training to promote knowledge about, and confidence to respond to, risky behaviours in adolescence. Findings indicated that the workshop was, overall, acceptable for participants, who reported higher levels of confidence to tackle risky behaviours in adolescence after attending the session. Teachers also reported greater levels of importance to tackle those issues following the workshop.

Overall, participants found the contents and methods of the workshops highly acceptable. They valued the skills-training component and the opportunity to engage in discussions with both peers and the group facilitators. Despite efforts to advertise the workshop widely to potential participants, only a limited number of individuals expressed interest in participating, and an even a smaller number completed the motivational ruler. Participants’ feedback indicated that this was largely due to the timing of the workshop (late afternoon) and organizational issues related to the school facilities. This underscores the need for greater collaborative efforts to identify strategies to increase access. There is a significant demand for the development and implementation of mental health prevention, early detection, and support programs that target caregivers and teachers’ needs. This is particularly important, as approximately 50% of all psychological disorders in adulthood begin by age 14 [38], and there has been a notable increase in prevalence rates, ranging from about 12% to more than 20%, of clinically severe anxiety and depression in youth cohorts following the COVID-19 pandemic [8]. Working together with carers and teachers to identify the best strategies to address their needs is a valuable opportunity to respond to this emergency.

The data on the short-term impact of the workshops on importance and confidence to respond to risky behaviours in adolescence were encouraging. Teachers reported a significant increase in both importance and confidence to respond to risky behaviours at the end of the workshop, and this change was associated with the baseline level of self-efficacy. It is possible that this type of intervention might be particularly beneficial for those with low levels of perceived self-efficacy. This is relevant when considering that teachers’ self-efficacy is linked to students’ outcomes (e.g., achievement and motivation) [39]. Carers reported a significant increase in their confidence to address risky behaviours, but not in importance. It is worth noting that carers, unlike teachers, expressed a high level of importance even before the workshop, as well as high self-efficacy, which could also explain why changes in importance and confidence to respond to risky behaviour were not associated with self-efficacy in this group. In this study, carers were all parents. Parents’ attitudes towards their loved ones’ mental health can vary depending on cultural, social, and individual factors. In many societies, there has been increasing awareness and recognition of the importance of mental health for adolescents, and parents are generally becoming more attuned to the mental health needs of their children, including teenagers [40]. The workshops in this study were attended on a volunteer basis and it is possible that a self-selected sample of parents participated. This adds to the poor generalizability of the findings, which is due to both the small sample size and its limited diversity (i.e., participants were predominantly Italian women, despite efforts to broaden recruitment to ethnic or other minorities; carers were all parents). Parent skills training might be perceived as “patronizing”, and fear of stigmatization, shame and guilt might prevent them from taking part. The stigma associated with seeking mental health support and access services for children is heightened especially among parents from ethnic minorities and those with children having special needs [41,42,43]. One possible solution to enhance participation and inclusion might be transitioning to online workshops, which could overcome spatial constraints, provide greater flexibility, and allow greater privacy [44]. The online workshops could also be integrated into guided self-help interventions (GSH), to facilitate the use of self-help materials [45] that carers and teachers could use in their own time, beyond the time and space constraints of standard therapies. Within the study presented, short video clips were developed for those who could not attend the workshops.

Nonetheless, despite these limitations, the findings of this study add to the evidence that providing families with knowledge and skills to cope with their children’s mental health difficulties is acceptable and associated with some benefit. For example, a recent systematic review of the literature on family support programs has demonstrated that these programs are effective in improving both caregivers’ and children’s mental health [46]. Similarly, the positive impact of the workshop found on teachers’ confidence and importance to tackle difficult behaviours in adolescents corroborates previous findings on the efficacy of teachers’ training to improve mental health literacy, reduce stigma, and increase confidence to offer help to students [47,48].

Future studies should include additional measures to assess a larger range of outcomes and identify groups who might especially benefit from interventions, such as the workshops tested in this study. These measures could, for example, assess teachers’ and parents’ stress (e.g., Teaching Stress Inventory [49], The Parental Stress Scale [50]), and adolescents’ strengths and difficulties (e.g., Strengths and Difficulties Questionnaire [51]), and might be repeated over time, to follow up on changes in attitudes and behaviours. It also seems appropriate to evaluate social desirability and use semi-structured interviews to enable participants to provide more comprehensive feedback.

## 5. Conclusions

This study represents a first attempt to evaluate a novel SSI combining psychoeducation and skill training for carers and teachers, with the aim of enhancing their knowledge and confidence to address risky behaviours in adolescence. Preliminary findings are promising and suggest that the workshops are acceptable and helpful in increasing participants’ confidence, particularly among those with lower self-efficacy.

The feasibility of (guided) self-help interventions for carers and teachers should be explored further, with a focus on increasing accessibility and sustaining benefits in the longer term. Working together with carers and teachers to identify the most effective intervention strategies is of paramount importance to meet these challenges.

## Figures and Tables

**Table 1:** Examples of vignettes and related questions.

**Teachers: Vignette 3**	
Luisa is 13 years old and she attends secondary school. She is a bright and high-achieving girl. She is well-integrated in her class and displays a positive attitude both towards her peers and teachers. Over the last few months, Luisa has avoided school at times when academic tests were due. Her goal is to succeed at school with very high marks. In order to achieve this goal, she believes it would be necessary to present herself well in high school. Despite receiving positive feedback from her teachers, Luisa often feels dissatisfied in herself. She tends to focus on little mistakes and perceives positive criticism in a negative way. She used to practice gymnastics at a competitive level. However, this year, she decided to give up this sport to be able to focus more on her studies. During parent–teacher meetings, teachers praise Luisa’s excellent abilities and her behaviour in class. Her parents believes that Luisa behaves very well at home, too. She spends most of her time at home studying, paying attention to even the smallest details until she achieves the top level. Luisa’s parents have noticed that she is worried about her academic career and that she suffers from stomach aches and headaches when a test is due. On a few occasions, they have allowed her to remain home and miss school.	How might Luisa be feeling? What behaviours are exhibited by Luisa? What does Luisa think about the situation? What perception might she have of herself? What factors have contributed to the situation and Luisa’s experiences? If no action took place, what could happen? On which factors could the teachers potentially intervene to help Luisa in this difficult situation?
**Carers: Vignette 1**	
Angela is a second-grade student in secondary school. Growing up, she has always been very extroverted and willing to play sports, causing no issues or concerns for her parents. At the parent–teacher meetings, the teachers report that Angela is hardworking and achieving good academic results. They also mention that, during recess, Angela tends to isolate from the rest of the class, often spending most of the time sitting at her desk, looking at her phone. Following the teachers’ feedback, Angela’s parents begin to observe her behaviour at home. They notice that she no longer goes out with her friends during the weekend and that she spends most of her time online on her phone. After a few weeks, during a family dinner, Angela starts sharing anecdotes about her friends. Her parents realize that they are unfamiliar with many of the names mentioned by her. They ask Angela about these friends, how she met them, and who they are. Angela says that they are friends she met through social media, with whom she shares common experiences and interests. Angela also says that she no longer wishes to participate in sports since she has never enjoyed doing so and that she has no interest in spending time outside.	What is the event to pay attention to? How might Angela be feeling? What behaviours are exhibited by Angela? What does Angela think about the situation? What perception might she have of herself? What factors have contributed to the situation and Angela’s experiences? If no action took place, what could happen? On which factors could carers potentially intervene to help Angela in this situation?

**Table 2:** Participants’ socio-demographic characteristics expressed as means (standard deviations) or frequencies (%).

Variables	Teachers		Carers	
	*N*	*M (SD)* or Frequency (%)	*N*	*M (SD)* or Frequency (%)
Age	28	50.64 (7.35) [min:33; max:64]	32	48.49 (5.54) [min:41; max:66]
Gender (Female vs. Male)	29	28 (96.55%)	32	27 (84.38%)
Nationality (Italian vs. Other)	29		32	
Italian		29 (100%)		30 (93.75%)
European		0 (0.00%)		1 (3.13%)
Extra-European		0 (0.00%)		1 (3.13%)
First Language (Italian vs. Other)	28	28 (100%)	32	30 (93.75%)
Level of Education	28		32	
Inferior to Diploma		0 (0.00%)		2 (6.25%)
Diploma		0 (0.00%)		5 (15.63%)
Bachelor’s Degree		1 (3.57%)		1 (3.13%)
Master’s Degree		21 (75.00%)		21 (65.63%)
Other title (e.g., conservatory, academy of fine arts)		4 (14.29%)		1 (3.13%)
Marital Status	27		32	
Single		3 (11.11%)		3 (9.38%)
Cohabiting		3 (11.11%)		6 (18.75%)
Married		16 (59.26%)		21 (65.63%)
Separated		3 (11.11%)		1 (3.13%)
Divorced		2 (7.41%)		0 (0.00%)
Widowed		0 (0.00%)		1 (3.13%)
Number of Children	25	1.32 (1.07)	32	2.00 (0.62)
Income (Euros)	22		27	
<15,000		2 (9.09%)		0 (0.00%)
15,000–29,000		12 (54.55%)		9 (33.33%)
30,000–55,000		7 (31.82%)		14 (51.85%)
56,000–100,000		1 (4.55%)		4 (14.82%)
Accommodation	26		30	
Owned house		21 (80.77%)		22 (73.33%)
Rented house		4 (15.39%)		6 (20.00%)
Other		0 (0.00%)		2 (6.67%)
Employment ^1^	-		32	
Full-time worker		-		14 (43.75%)
Part-time worker		-		10 (31.25%)
Self-employed		-		6 (18.75%)
Homemaker		-		1 (3.13%)
Unemployed (actively seeking employment)		-		1 (3.13%)
Years of teaching ^2^	29	18.21 (9.16) [min:1.00–max:36.00]	*-*	*-*
Hours of teaching (per week) ^2^	28	16.43 (5.53)	*-*	*-*
Number of classes ^2^	28	4.75 (4.07)	*-*	*-*
Students with special needs (Yes vs. No) ^2^	26	24 (92.31%)	*-*	*-*
Psychological Difficulties (Yes vs. No)	27	3 (11.11%)	32	5 (15.63%)
Medical Illnesses (Yes vs. No)	27	4 (14.81%)	32	3 (9.38%)
DASS-21 total score	29	10.31 (7.15)	31	11.58 (10.24)
TSES-SF ^2^ total score	29	6.60 (1.01)	-	-
PSOC ^1^ total score	-	-	30	64.07 (9.41)

Notes. DASS-21 = Depression, Anxiety and Stress Scale—21; TSES = Teachers’ Sense of Efficacy Scale-Short Form; PSOC = Parenting Sense of Competence. ^1^ Variables collected only for carers. ^2^ Variables collected only for teachers.

**Table 3:** Qualitative feedback on the workshop.

Variables	Teachers		Carers	
	*N*	Frequency (%)	*N*	Frequency (%)
Clarity	26		27	
Not at all		0 (0.00%)		0 (0.00%)
Somewhat		2 (7.69%)		5 (18.52%)
Very		19 (73.08%)		14 (51.85%)
Extremely		5 (19.23%)		8 (29.63%)
Utility	26		27	
Not at all		0 (0.00%)		0 (0.00%)
Somewhat		6 (23.08%)		9 (33.33%)
Very		17 (65.39%)		14 (51.85%)
Extremely		3 (11.54%)		4 (14.82%)
Materials’ suitability	26		26	
Not at all		0 (0.00%)		0 (0.00%)
Somewhat		4 (15.39%)		6 (23.06%)
Very		17 (65.39%)		13 (50.00%)
Extremely		5 (19.23%)		7 (26.93%)
Engagement	26		27	
Not at all		0 (0.00%)		0 (0.00%)
Somewhat		2 (7.69%)		2 (7.41%)
Very		21 (80.77%)		19 (70.37%)
Extremely		3 (11.54%)		6 (22.22%)
Strengths	26		23	
Skills-training/vignettes		20 (76.92%)		18 (78.26%)
Speakers’ competence		9 (34.62%)		3 (13.04%)
Neurobiological insights		2 (7.69%)		0 (0.00%)
Weaknesses	26		23	
Lack of practical strategies		5 (19.23%)		1 (4.35%)
Short duration		7 (26.92%)		6 (26.09%)
Poor attendance		2 (7.69%)		6 (26.09%)
Suggested topics	26		23	
Specific disorders (e.g., eating disorders, learning disorders, disabilities)		2 (7.69%)		1 (4.35%)
Group interactions		8 (30.77%)		4 (17.39%)
New technologies and social media		2 (7.69%)		10 (43.48%)
School-family relationships		2 (7.69%)		3 (13.04%)

**Table 4:** Wilcoxon matched-pairs signed rank tests.

***Teachers*** ^1^					
Motivational ruler	Pre *M (SD)*	Post *M (SD)*	z	*p*-value	*r*_rb_
Importance	7.81 (1.07)	8.15 (1.09)	2.053	0.021	0.524
Confidence	7.65 (0.89)	8.35 (1.02)	3.615	<0.001	0.900
***Parents*** ^2^					
Motivational ruler	Pre *M (SD)*	Post *M (SD)*	z	*p*-value	*r*_rb_
Importance	9.10 (1.12)	9.29 (1.02)	0.700	0.241	0.306
Confidence	7.86 (1.22)	8.69 (1.00)	2.981	0.002	0.974

Notes. Importance and confidence to address adolescents’ risky behaviours assessed using motivational rulers (Visual Analogue Scale from 0 = “Not at all” to 10 = “Extremely”). ^1^ N = 24. ^2^ N = 14.

## Data Availability

The data presented in this study are available on request from the corresponding author. The data are not publicly available due to they report private information about participants.
